# Relationship between gut microbiome characteristics and the effect of nutritional therapy on glycemic control in pregnant women with gestational diabetes mellitus

**DOI:** 10.1371/journal.pone.0267045

**Published:** 2022-04-15

**Authors:** Jing Chen, Yuying Yang, Ningning Yu, Wanxiao Sun, Yuanyuan Yang, Mei Zhao

**Affiliations:** 1 School of Nursing, Anhui Medical University, Hefei, Anhui Province, The people’s Republic of China; 2 Division of Life Sciences and Medicine, Department of Nursing, Hefei Ion Medical Center, The First Affiliated Hospital of USTC, University of Science and Technology of China, Hefei, Anhui Province, The people’s Republic of China; 3 Department of Obstetrics and Gynecology, The First Affiliated Hospital of Anhui Medical University, Hefei, Anhui Province, The people’s Republic of China; University of Hawai’i at Manoa College of Tropical Agriculture and Human Resources, UNITED STATES

## Abstract

The purpose of this study was to explore the relationship between the characteristics of gut microbiome and the effect of medical nutrition therapy (MNT) on glycemic control in pregnant women with gestational diabetes mellitus (GDM). Seventy-four pregnant women newly diagnosed with GDM received MNT for one-week. The effect of glycemic control was evaluated by fasting and 2-hour postprandial blood glucose; and stool samples of pregnant women were collected to detect the gut microbiome before and after MNT. We used a nested case-control study design, with pregnant women with GDM who did not meet glycemic standards after MNT as the ineffective group and those with an age difference of ≤5 years, matched for pre-pregnancy body mass index (BMI) 1:1, and meeting glycemic control criteria as the effective group. Comparison of the gut microbiome characteristics before MNT showed that the ineffective group was enriched in *Desulfovibrio*, *Aeromonadales*, *Leuconostocaceae*, *Weissella*, *Prevotella*, *Bacillales_Incertae Sedis XI*, *Gemella* and *Bacillales*, while the effective group was enriched in *Roseburia*, *Clostridium*, *Bifidobacterium*, *Bifidobacteriales*, *Bifidobacteriaceae*, *Holdemania* and *Proteus*. After treatment, the effective group was enriched in *Bifidobacterium* and *Actinomycete*, while the ineffective group was enriched in *Holdemania*, *Proteus*, *Carnobacteriaceae* and *Granulicatella*. In conclusion, the decrease in the abundance of characteristic gut microbiome positively correlated with blood glucose may be a factor influencing the poor hypoglycemic effect of MNT in pregnant women with GDM. Abundance of more characteristic gut microbiome negatively correlated with blood glucose could help control blood glucose in pregnant women with GDM.

## Introduction

Gestational diabetes mellitus (GDM) is the first identification or occurrence of different degrees of impaired glucose tolerance during pregnancy, which is a common complication of pregnancy. According to the diagnostic guidelines recommended by the International Diabetes and Pregnancy Research Organization (IADPSG) [1], the incidence of GDM is as high as about 18% in the U.S. A systematic study indicated that the overall prevalence of GDM in the Chinese population was 14.8% [2]. In addition, an increasing number of pregnant women worldwide are being diagnosed with GDM. Prevention and treatment of GDM have received widespread attention. Untreated or poorly treated GDM may result in the inability of body to maintain normal glucose metabolism, with significant impact on maternal and infant health, including increased incidence of caesarean section, shoulder dystocia, birth injuries, major bleeding, and increasing the risk of large infants, intrauterine distress, and small-for-gestational age infants [3,4]. Moreover, pregnant women with GDM who have poor glycemic control are at increased risk of postpartum type 2 diabetes and their offspring are more likely to suffer from obesity and metabolic syndrome [5,6]. Therefore, it is important to actively and scientifically control the blood glucose of pregnant women with GDM.

The guidelines for GDM explicitly recommends that pregnant women with GDM should begin medical nutrition therapy (MNT) for a period of one-week [7]. If fasting blood glucose (FBG) is higher than 5.1mmol/L or two-hour postprandial blood glucose is higher than 6.7 mmol/L after MNT, pharmacotherapy should be considered [8]. The first-line drug recommended by the guidelines is insulin. However, factors such as the high cost of multiple injections over a long period of time and injection-induced the pain caused by injections in pregnant women with GDM will lead to poor compliance with insulin therapy and may lead to complications such as hypoglycemia and overweight [9]. In addition, some guidelines do not recommend the use of oral hypoglycemic agents during pregnancy because there is insufficient evidence in population studies to demonstrate the safety of oral hypoglycemic agents during pregnancy [10]. Moreover, studies in recent years have shown that self-efficacy, economic status, occupation, age, and other social factors will influence the effectiveness of glycemic control [11]. However, auxiliary therapy from these perspectives cannot effectively improve the effect of glycemic control in patients with GDM. In practical terms, about 30% - 40% of pregnant women with GDM have poor response to one-week MNT, and the effect of MNT on blood glucose control needs to be enhanced [12]. Therefore, due to the limitations of hypoglycemic pharmacotherapy and auxiliary glycemic control measures during pregnancy mentioned above, how to improve the effect of glycemic control in pregnant women with MNT is a momentous problem that deserves further exploration.

Recent studies have shown that the relationship between gut microbiome and blood glucose levels and its impact on hypoglycemic therapy is noteworthy. At present, probiotic preparations have developed rapidly in the fields of regulating microecology and adjuvant treatment of related diseases, and their safety has also been guaranteed. A randomized, double-blind and placebo-controlled clinical trial showed taking probiotics containing Lactobacillus and Bifidobacterium for 12 weeks could reduce FBG levels in diabetic patients [13]. A female cohort study comparing the gut microbiome of women with normal glucose tolerance, impaired and diabetic glucose control revealed an increase in Clostridium species in the gut of women with type 2 diabetes, which was negatively correlated with FBG, glycosylated hemoglobin, and insulin levels, whereas Lactobacillus species in the gut were positively correlated [14]. Studies have shown that pregnant women with GDM have gut microbiome imbalance compared to normal pregnant women [15,16], with decreased numbers of Bifidobacterium, Lactobacillus, and Bacteroides in the intestine, and an increase in the number of Enterobacteria and Yeast. The reduction of Bifidobacterium and Bacteroides is not conducive to the body’s lipid metabolism and easily causes insulin resistance [17]. Previous studies have focused on the preventive effects of probiotics on GDM. A clinical study with a probiotic strain intervention during the first trimester showed a significant reduction in the incidence of GDM in the intervention group [18]. At present, several randomized controlled trials have found that taking probiotics during pregnancy can improve insulin resistance and reduce blood glucose level [19,20]. These studies confirmed that the body’s blood glucose status is affected by the characteristics of gut microbiome. It has been reported that short-term dietary adjustment can change the structure of gut microbiome, gut microbiome activity and gene expression [21]. To some extent, the characteristics of gut microbiome may be an important factor influencing the effect of glycemic control after MNT in pregnant women with GDM.

However, there are no studies focused on changes in the characteristics of gut microbiome in pregnant women with GDM before and after MNT, or in pregnant women with poor glycemic control. Thus, this goal of study was to analyze the changes in gut microbiome characteristics in pregnant women with GDM before and after MNT and to explore the relationship between the gut microbiome characteristics and the glycemic control effect before and after MNT in pregnant women with GDM, in order to clarify the gut microbiome’s close relationship to MNT hypoglycemic effect and to provide a reference for a targeted gut microbiome intervention program.

## Materials and method

### Study design and subjects

A nested case-control study design was adopted in this study. Between July 2018 and May 2019, 120 pregnant women with a first diagnosis of GDM were recruited from the obstetrics clinic of the First Affiliated Hospital of Anhui Medical University for regular obstetric examinations. All participants signed an informed consent. This study was approved by the ethics committee of Anhui Medical University.

The inclusion criteria were: (1) diagnosed as GDM for the first time; (2) 24–28 weeks of pregnancy; (3) within one-week of GDM confirmed by oral glucose tolerance test (oral glucose tolerance test, OGTT); (4) singleton pregnancy; (5) normal expression and understanding ability. Pregnant women who had the following criteria were excluded: (1) prediabetes or diabetes mellitus, hypertension, thyroid disease, asthma, lipid metabolism disorders, inflammatory bowel disease, irritable bowel syndrome and celiac disease were present before pregnancy or after being included in this study; (2) antibiotics (penicillin, amoxicillin, cephalosporin antibiotics, etc.) have been taken after 20 weeks of pregnancy; (3) having taken probiotics (*Lactulose*, *Pefikang*, *Bifidobacteria*, etc.) after 20 weeks of pregnancy; (4) with incomplete inspection records and stool specimen collection during the study.

All subjects in this study received MNT for one-week under the professional guidance of the chief physician and the researcher. The guidelines [22,23] clearly pointed out that it is necessary to observe whether the pregnant women with GDM can control blood glucose effectively after one-week interval of MNT. Drug treatment can also be given in time to ensure the safety of pregnant women and fetuses for pregnant women with GDM who cannot control blood glucose effectively after one-week interval of MNT. According to the criterion of MNT from GDM guidelines [24], we provided nutritional education and nutritional counseling to each pregnant woman with GDM and developed individualized recipes based on their height, pre-pregnancy body mass index (BMI), preferences, education level, as well as computing ability.

1. The daily serving number was calculated according to the food exchange portion methods.
2. The proportion of carbohydrates was 50–60%, and the remaining energy supplying nutrients contained 15–20% protein and 25–30% fat, with a reasonable distribution of cereals and potatoes, eggs, legumes, fish, dairy products, vegetables and oils.
3. The portions of cereals and potatoes, egg, bean, fish and vegetables were evenly distributed over three meals, while milk and products were distributed over 2–3 additional meals.

The daily dietary intake shall meet the following conditions: the recommended ratio of coarse grain staple food to fine grain staple food was 1:3; vegetables were mainly green leafy vegetables, with appropriate amount of rhizomes, eggplant fruits and fungi; the cooking method was less oil and salt. Moreover, it is obligatory for pregnant women with GDM to maintain 30 minutes of moderate-intensity aerobic exercise at least 5 days a week. The recommended exercise methods were stair-climbing exercises and power walking.

During one-week of MNT, 25 subjects were failed to comply with the overall process of MNT, or they did not follow the principles of diet and exercise in MNT, and 21 subjects were lost to follow-up. Finally, 74 pregnant women were included in this study.

### Data collection

Subjects were recruited by completing a general information questionnaire that included age, education level, whether they were passive smoker, economic income, gravida, parity, history of hyperemesis gravidarum, history of delivery of macrosomia, history of delivery of low-birth-weight infants, and family history of diabetes, body mass index (BMI), etc. Passive smoking was defined as exposure to smoke from smokers for at least 15 minutes per day and >1 day per week among non‐smokers [25].

To ensure the therapy compliance of the subjects, the pregnant women were informed by telephone on the day of diagnosis to attend obstetric clinic on the same day or the next day. The expert and researchers worked together to provide dietary guidance and establish management of Wechat platform. The subjects were required to record their daily dietary intake in detail, and upload photos of all food intake to the WeChat platform for dietary assessment. They also typed up a daily exercise log on the WeChat platform. Researchers should provide timely feedback and adjust the subjects’ dietary intake and record it.

### The standards for blood glucose control

One-week later, the fasting and two-hour postprandial blood glucose levels were measured. At the end of one-week of MNT, the blood glucose profile was used to evaluate the effect of glycemic control.FBG<5.1mmol/L, one-hour postprandial blood glucose<7.8mmol/L, and two-hour postprandial blood glucose<6.7mmol/L are the blood glucose standard conditions; reaching all of these criteria means glycemic management was up to standard [6], otherwise it was not up to standard.

### Matching method

A nested case-control study design was adopted, in which pregnant women with GDM who did not meet the glycemic control results after one-week of using MNT were considered as the ineffective group (group N), and pregnant women with GDM whose age difference was less than 5 years and whose BMI was at the same level as that of the pregnant women who met the glycemic standard were considered as the matching requirement, and the number of effective groups (group Y) was matched 1:1.The Y group consisted of pregnant women with GDM whose glycemic control effect was on target and was separated into Y1 and Y2 based on before and after therapy, respectively. The N group was classified into N1 and N2 based on before and after therapy.

### Sample collection and DNA extraction

Stool samples from subjects before and after MNT were collected uniformly by trained study personnel. We distributed disposable sterile bowls for stool collection to the subjects beforehand and introduced points to note. Subjects picked up approximately 1 g of stool with a small spoon after a natural bowel movement. Fresh stool samples were collected from recruited subjects and transported to the laboratory in ice packs within 2 hours. All samples were then frozen immediately and stored at—80°C prior to analyses.

DNA was extracted from each fecal samples using improved protocol based on the manual of QIAamp Fast DNA Stool Mini Kit (Qiagen, Germany). In detail, 1ml of InhibitEX Buffer and proper amount of glass beads (0.5mm diameter, Qiagen) was added to each 200mg of feces. The mixture was homogenized and beat with 60Hz for 1 min twice with a Homogeneous instrument (FASTPREP-24, Aosheng Biotech, China). Afterwards, the DNA purification was performed according to the manufacturer’s instructions.

### 16S rRNA amplicon sequencing

HiSeq/MiSeq platform PE250 strategy (Illumina, Inc., CA, USA) was used for double terminal sequencing, and PANDAseq software was used to produce the high-quality assemblies from Illumina paired-end reads.

The V3-V4 region of the bacteria 16S ribosomal RNA genes were amplified by PCR using barcoded primers 341F 5’-CCTACGGGRSGCAGCAG-3’ and 806R 5’-GGACTACVVGGGTATCTAATC-3’. Negative controls consisted of empty sterile storage tubes for DNA extraction and amplification using the same procedures and reagents as for the fecal samples.No amplification was detected in the negative controls.

### Bioinformatics processing and statistical analysis

Assembled tags, trimmed of barcodes and primers were further checked on their rest lengths and average base quality. The 16S sequence were restricted between 220 bp and 500 bp to ensure the average quality value of each reads was not less than 20 and the number containing N was not exceed three. Operational Taxonomic Units (OTUs) were clustered based on 97% similarity through using UPARSE and chimeric sequences were identified and removed through Usearch (version 7.0.1090). Each representative sequence was assigned to a taxa by RDP Classifer against the RDP database setting confidence threshold to 0.8. The copy number of sequences was enumerated, and redundant parts of the repeated sequences were removed. Only the sequences with frequency more than 1 (which tend to be more reliable) were clustered into OTUs, each of which had a representative sequence. OTU profiling table and alpha diversity analyses were also achieved by python scripts of QIIME (version 1.9.1).

Alpha diversity assessed by the Shannon index and Simpson index were used to estimate gut microbiota community richness. The value of alpha diversity index of the samples was calculated by QIIME software. Beta diversity analysis was conducted to evaluate the gut microbiome differences in species diversity between sample groups. The beta diversity among the N/Y groups were calculated by unweighted and weighted UniFrac distances and illustrated by principal coordinates analysis (PCoA) plots. Linear discriminant analysis (LDA) Effect Size (LEfSe) was performed to estimate the impact of each species abundance on the differential effect and identify the communities or species that have significant differences effect in sample division.

The data were analyzed by SPSS 21.0 statistical software. In the clinical case data, normal distributed continuous variables were described as mean and standard deviation, and the non-normal distribution continuous variables were described as the median and interquartile range. Statistical inference group independent-sample t-test was used for inter-comparison; frequency and percentile were used for statistical description of counting data, and chi-square test was used for inter-group comparison. The gut microbiome parameters were tested by rank sum test. A *P* value less than 0.05 was considered statistically significant.

## Results

### General characteristics of the pregnant women

A total of 74 pregnant women newly diagnosed with GDM completed an one-week MNT and recorded a diet diary. After one-week of MNT, 12 subjects failed to meet the standard of glycemic control, and 62 subjects met the standard. There was no significant difference in age, pre-BMI, weight gain during pregnancy, education level, passive smoker, economic income, pregnancy frequency, parity, abortion, hyperemesis gravidarum and family history of diabetes mellitus between the two groups (Table 1).

**Table 1:** Comparison of the basic demography, gestational history and pregnancy outcome of pregnant women in the blood glucose control effective group and ineffective group.

General characteristics		Effective group (n = 62)		Ineffective group (n = 12)		*t/x* ^*2*^	*P*
		Mean	SD	Mean	SD		
Age (y)		30.2	4.9	32.2	4.1	1.393	0.168
Pre-BMI (kg/m^2^)		21.7	2.2	23.0	3.3	1.296	0.218
Weight gain (kg)		11.9	2.0	12.2	1.6	0.495	0.622
Education (n (%))						0.802	0.938
Junior		6(9.7)		1(8.3)			
High school/technical secondary school		10(16.1)		2(16.7)			
College/ Vocational College		15(24.2)		4(33.3)			
Undergraduate		21(33.9)		4(33.3)			
Postgraduate and above		10(16.1)		1(8.3)			
Passive smoker (n (%))						0.414	0.52
	Yes	45(72.3)		7(58.3)			
	No	17(27.4)		5(41.7)			
Monthly income (CNY)						1.66	0.646
	<5000 (n (%))	10(16.1)		1(8.3)			
	5000~ (n (%))	16(25.8)		2(16.7)			
	7000~ (n (%))	16(25.8)		5(41.7)			
	≧9000 (n (%))	20(32.3)		4(33.3)			
Gravida (n (%))						2.846	0.241
	1	37(59.7)		4(33.3)			
	2	15(24.2)		5(41.7)			
	≧3	10(16.)		3(25.0)			
Parity (n (%))						0.719	0.396
	Primipara	42(67.7)		6(50.0)			
	Multipara	20(32.2)		6(50.0)			
Abortions (n (%))						3.178	0.204
	0	51(82.3)		8(66.7)			
	1	4(6.5)		3(25.0)			
	≧2	7(11.3)		1(8.3)			
Hyperemesis gravidarum (n (%))						0.07	0.792
	Yes	21(33.9)		3(25.0)			
	No	41(66.1)		9(75.0)			
Family diabetes (n (%))						0.403	0.525
	Yes	8(12.9)		3(25.0)			
	No	54(87.1)		9(75.0)			

SD: Standard deviation.

### Comparison of glycemic results in pregnant women with GDM before and after MNT

The comparison of FBG, 1-hour and 2-hour postprandial blood glucose levels between the two groups before MNT and FBG, 2-hour postprandial blood glucose levels after MNT is shown in Tables 2 and 3. The 1-hour postprandial blood glucose level in ineffective group was significantly higher than that of effective group before MNT (*P* < 0.05). The FBG and 1-hour postprandial blood glucose level in effective group was significantly lower than those in the ineffective group after MNT (*P* < 0.05).

**Table 2:** Comparison of OGTT before MNT in the blood glucose control effective group and the ineffective group.

OGTT	Effective group (n = 62)		Ineffective group (n = 12)		*t*	*P*
	Mean	SD	Mean	SD		
FBG (mmol/L)	5.19	1.23	5.67	0.94	1.256	0.213
1h blood-glucose (mmol/L)	8.19	2.94	10.73	2.15	2.030	0.046
2h blood-glucose (mmol/L)	8.29	1.89	9.41	1.43	1.939	0.057

FBG: Fasting blood glucose.

**Table 3:** Comparison of glycemic results after MNT in the blood glucose control effective group and the ineffective group.

Objects	Effective group (n = 62)		Ineffective group (n = 12)		*t*	*P*
	Mean	SD	Mean	SD		
FBG (mmol/L)	4.58	0.29	5.16	0.62	3.159	0.008
2h blood-glucose (mmol/L)	5.46	0.59	7.04	1.42	3.783	0.003

FBG: Fasting blood glucose.

### Comparison of gut microbiome in pregnant women with GDM before therapy

**Fig 1:**
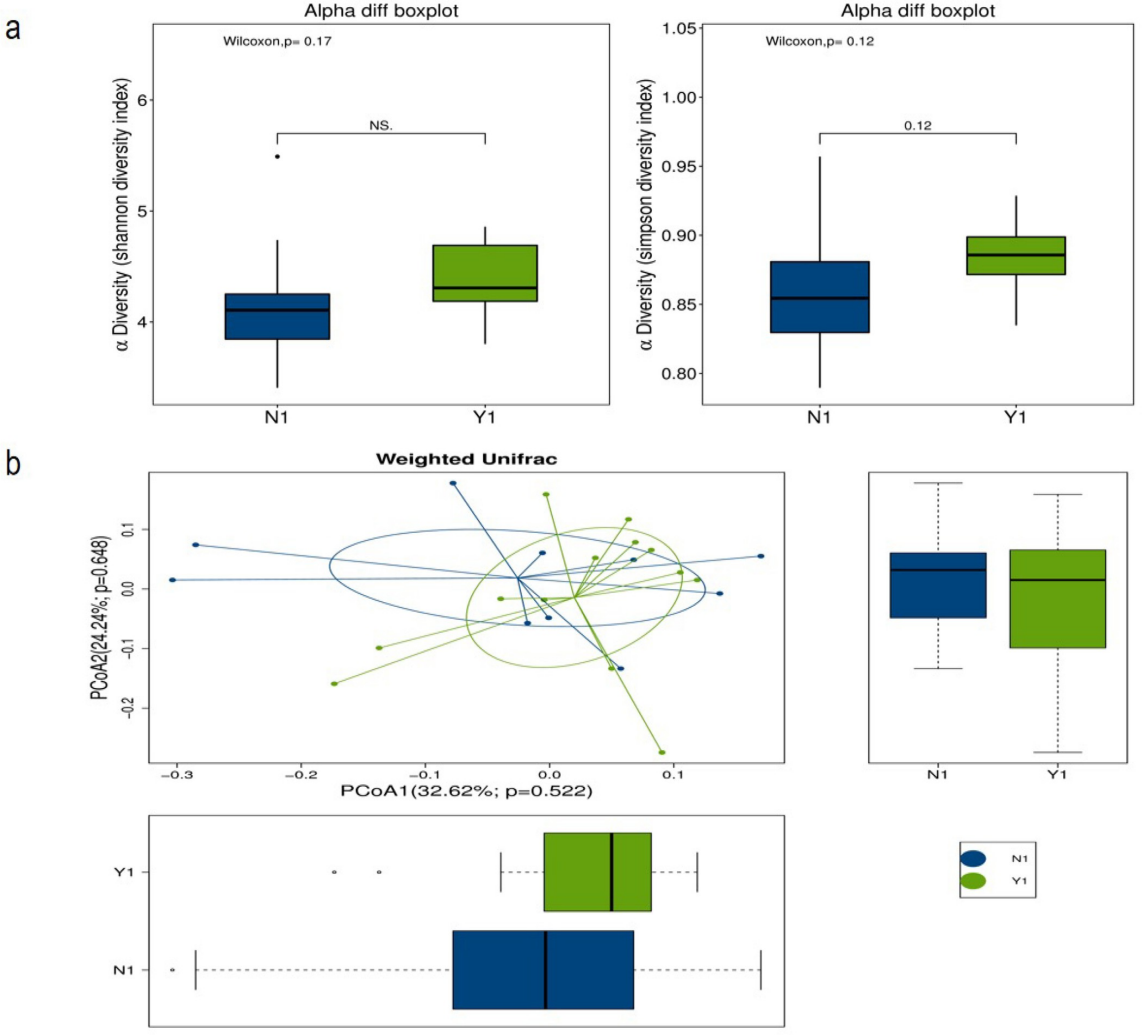
The alpha diversity and beta diversity for the effective group and ineffective group before the therapy. (a) Alpha diversity between the effective group and the ineffective group before the therapy; (b) Beta diversity between the effective group and the ineffective group before the therapy: Weighted Unifrac distance.

The gut microbiome of pregnant women with GDM before and after therapy were compared between effective group and ineffective group after 1:1 matching. Alpha diversity of N_1_ and Y_1_ groups is shown in Fig 1A. Before MNT, the biodiversity of effective group was higher than that of ineffective group, but the Shannon index and Simpson index of the two groups were not statistically different. The beta diversity of gut microbiome was not significantly different between the two groups according to the weighted UniFrac distance (Fig 1B).

**Fig 2:**
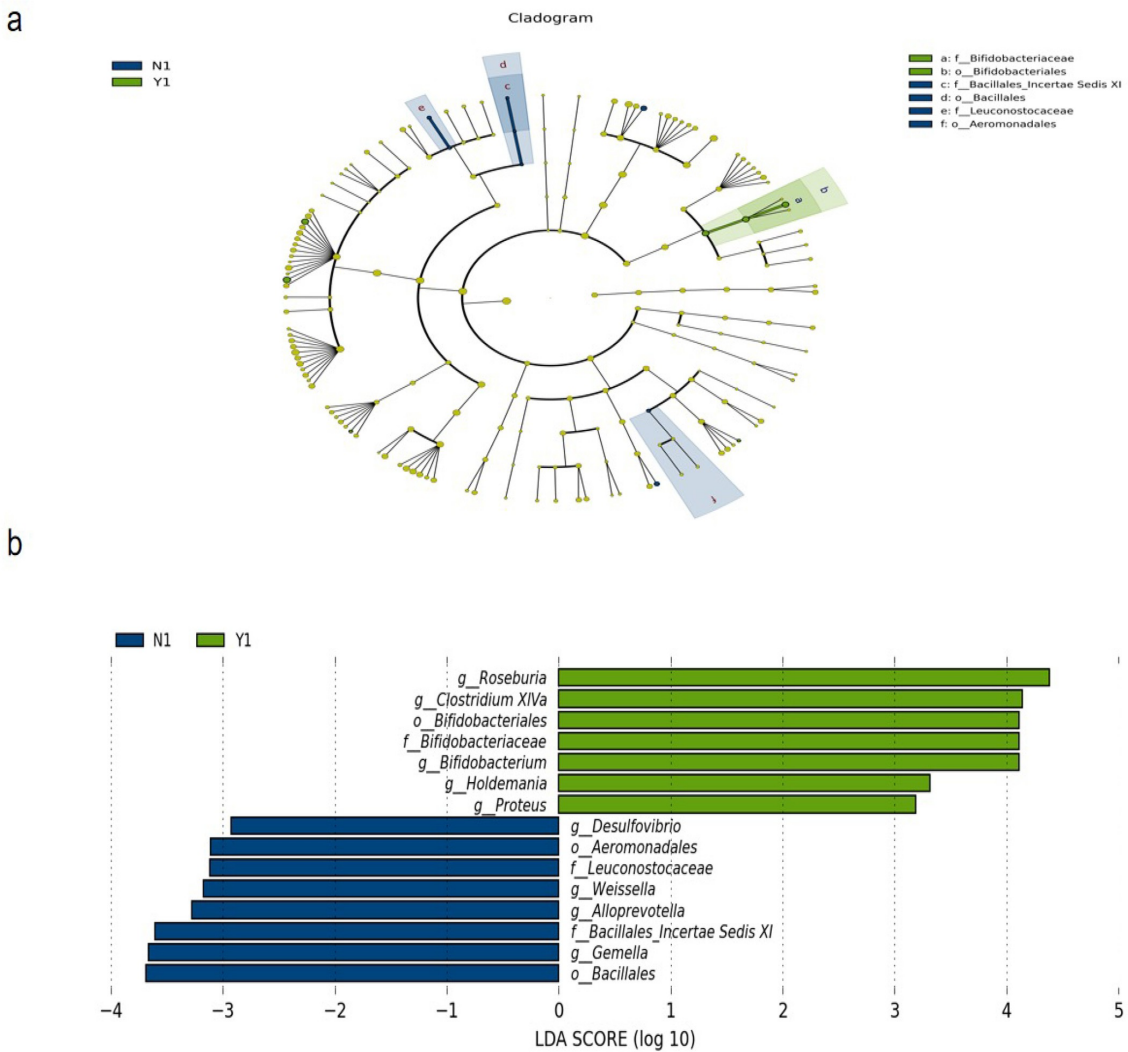
The results of LEfSe (LDA Effect Size) between the effective group and the ineffective group before the therapy. (a) The evolutionary branching graph showed the differences in species richness between the two groups, the circle radiating from inside to outside in the figure represents the taxonomic level from phylum to genus. The species with significant differences are colored with the group. The green node indicates the microbial groups that play an important role in the effective group, and the blue node indicates the microbial groups that play an important role in the ineffective group. (b) The histogram of the distribution of LDA values mainly shows species with significantly different LDA scores greater than a predetermined value, i.e. biomarkers that are statistically different between the effective and ineffective groups before MNT.

As shown in Fig 2A, the differences in species richness between the two groups were mainly *Bifidobacteriaceae and Bifidobacteriales* in the effective group and *Bacillus_Incertae Sedis XI*, *Bacillales*, *Leuconostocaceae*, and *Aeromonadales* in the ineffective group. Further statistical analysis on the LDA scores of these groups showed that *Roseburia*, *Clostridium*, *Bifidobacterium*, *Bifidobacteriales*, *Bifidobacteriaceae*, *Holdermania* and *Proteus* were enriched in the effecitve group, while *Desulfovibrio*, *Aeromonadales*, *Leuconostocaceae*, *Weissella*, *Prevotella*, *Bacillales_Incertae Sedis XI*, *Gemella* and *Bacillales* were enriched in the ineffective group (Fig 2B).

### Changes in gut microbiome before and after MNT

**Fig 3:**
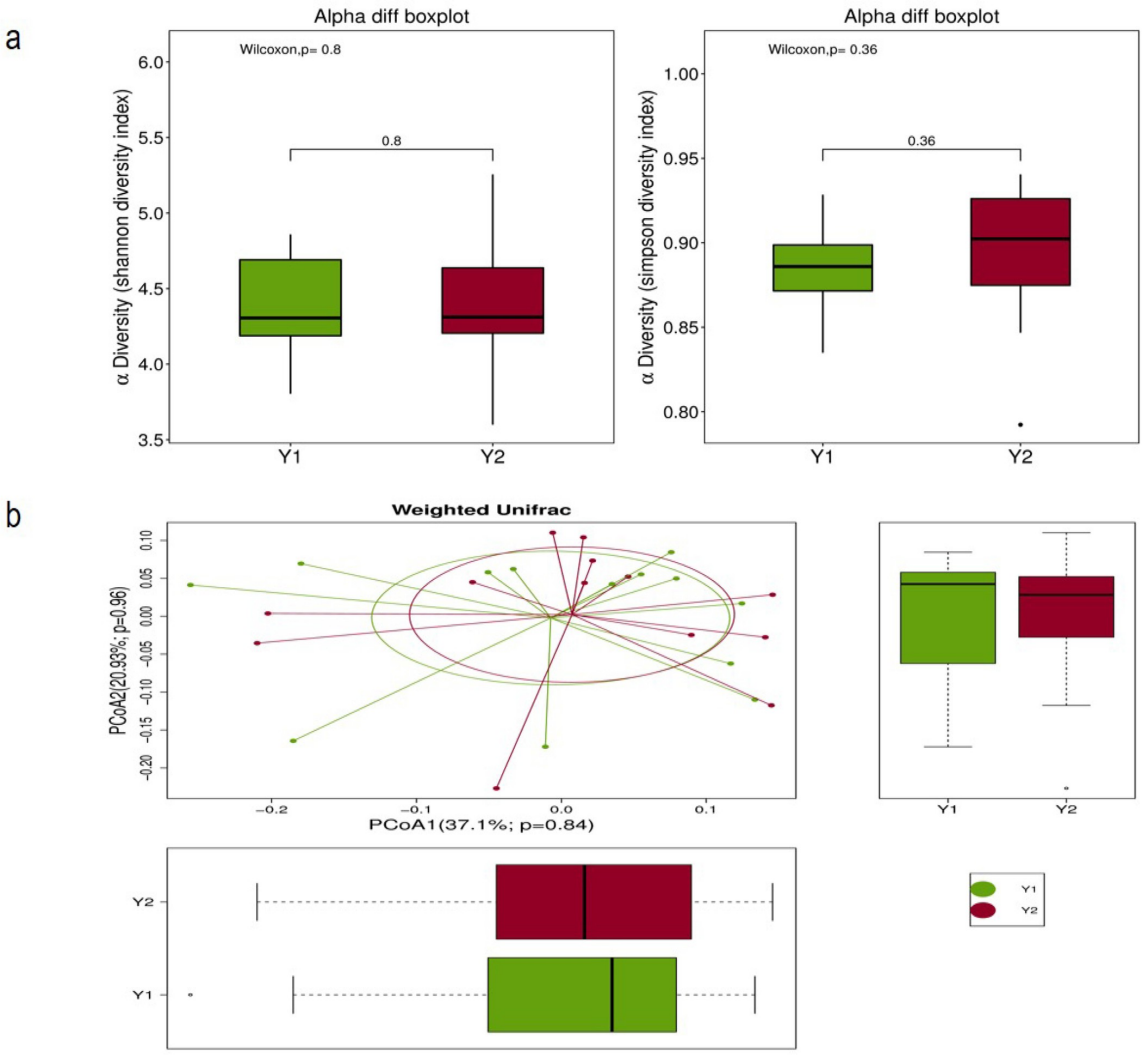
Changes in the gut microbiome of the effective group before and after MNT. (a) Alpha diversity between the effective group before and after therapy; (b) Beta diversity between the effective group before and after therapy: Weighted Unifrac distance.

**Fig 4:**
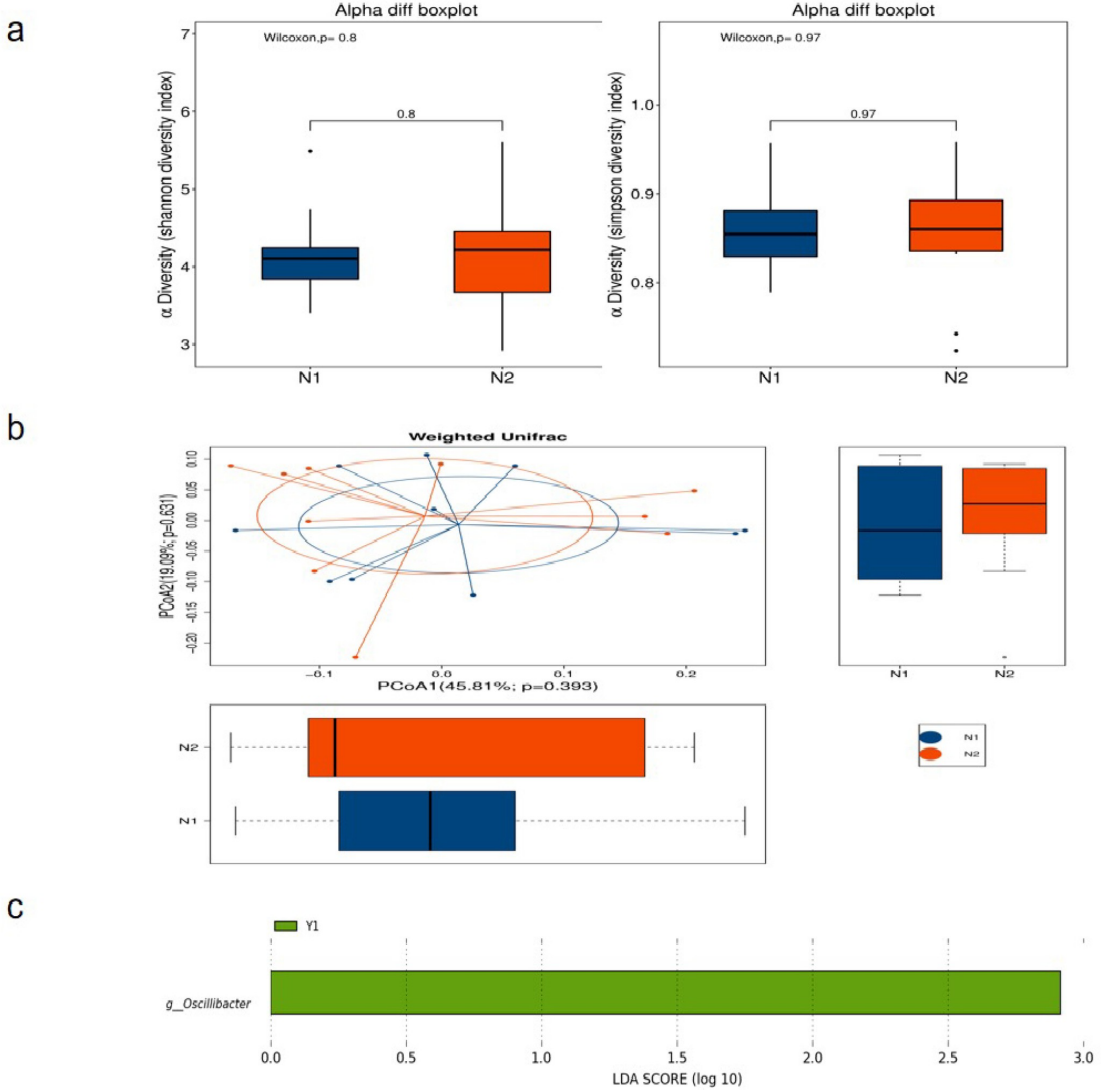
Changes in the gut microbiome of the ineffective group before and after MNT. (a) Alpha diversity between the ineffective group before and after therapy; (b) Beta diversity between N1 and N2 group: Weighted Unifrac distance; (c) Results of linear discriminant analysis of gut microbiome before and after therapy outcome between effective group before and after therapy.

After one-week MNT, alpha diversity (Simpson diversity index) of the effective group showed an increasing trend but was not statistically significant. There was an increasing trend, but no statistical significance, and no significant change in the beta diversity of the gut microbiome (Fig 3). The alpha diversity and beta diversity of the gut microbiome in the ineffective group did not change significantly before and after MNT (Fig 4A and 4B). Further species difference analysis showed that there was no species difference in the ineffective group before and after MNT, however, the abundance of *Oscillatoria* in the gut microbiome of the effective group was significantly reduced after MNT nevertheless (Fig 4C).

### Comparison of gut microbiome in pregnant women with GDM after MNT

**Fig 5:**
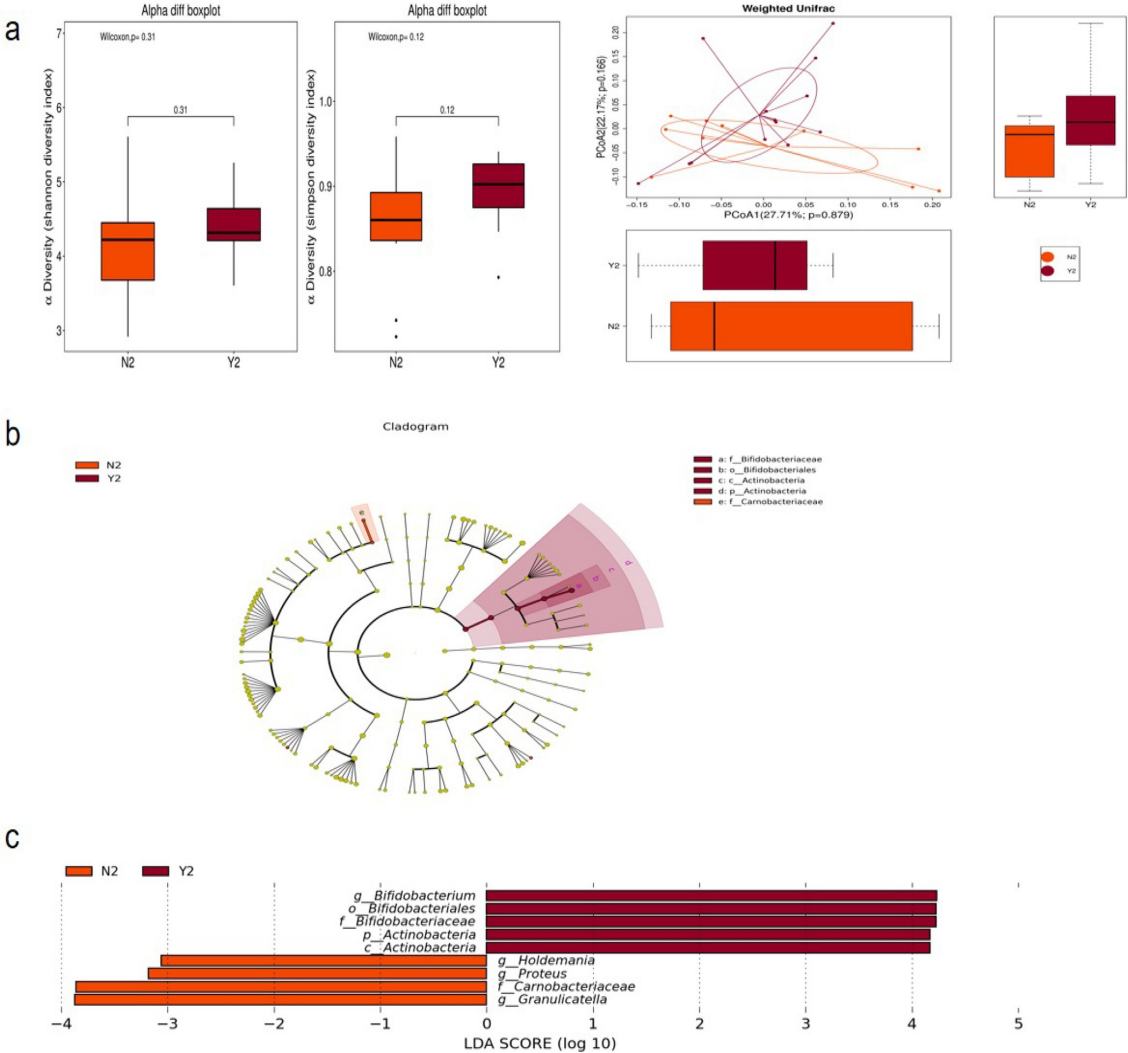
Comparison of gut microbiome in pregnant women with GDM after MNT. (a). Alpha diversity between the effective group and the ineffective group after the therapy; beta diversity between the effective group and the ineffective group after the therapy: Weighted Unifrac distance; (b-c). LEfSe outcome between the effective group and the ineffective group after the therapy.

After MNT, the alpha diversity index of the effective group appeared to be higher than that of the ineffective group, but Shannon index and Simpson index were not statistically different between the two groups (Fig 5A). The first and second principal coordinates allowed discrimination between the two groups, but the difference was not statistically significant after weighting N_2_ and Y_2_ groups (Fig 5A). The species abundance differences between the two groups were mainly derived from *Bifidobacteriaceae*, *Bifidobacteriales* and *Actinobacteria* in Y_2_ group and *Carnobacteruaceae* family in N_2_ group (Fig 5B). Further statistics on the LDA scores of these groups were shown in Fig 5C, showing that *Bifidobacterium*, *Bifidobacteriales*, *Bifidobacteriaceae* and *Actinobacteria* were enriched in the effective group, while *Holdemania*, *Proteus*, *Carnobacteriaceae* and *Granulicatella* were enriched in the ineffective group. The different species that were more abundant in group N and group Y were showed in Table 4.

**Table 4:** Comparison of different species enriched in effective groups and ineffective groups.

Species	Role in metabolism	Group N1	Group N2	Group Y1	Group Y2
*Roseburia*	*Roseburia* plays a role in metabolic reprogramming, immune activation, and in sustaining the gut barrier. *Roseburia* spp. are a critical butyrate-producing bacteria cluster. The potential role of butyrate is by inhibiting histone deacetylase (histone deacetylase, HDAC) or interacting with G protein-coupled receptors (G protein-coupled receptors, GPCRs) such as free fatty acid receptors 2 (FFAR2) and 3 (FFAR3) in the control of body weight and insulin sensitivity [26]. Studies have uncovered the dysbiosis microbiome profile of (inflammatory bowel disease, IBD) with significantly low numbers of *Roseburia* and revealed a low number of *Roseburia* intestinalis in (Crohn’s disease, CD) patients [27,28]. The abundance of *Roseburia* showed a decreasing trend in T2DM patients [29]. Moreover, studies found that *Roseburia* was positively correlated with BMI [30].			*	
*Clostridium*	*Clostridium* innocuous has recently been identified as the pathogen of antibiotic associated diarrhea in humans [31].			*	
*Bifidobacterium*	*Bifidobacterium* population has a beneficial effect on the intestinal environment of newborns undergoing cesarean section, which is closer to that of newborns undergoing vaginal delivery, especially in terms of colonization[32].Studies have shown that pregnant women with GDM have gut microbiome imbalance compare to normal pregnant women, with decreased numbers of *Bifidobacterium*[33,34].			*	*
*Bifidobacteriales*	Compared with neonates without jaundice, *Bifidobacteriaceae* were decreased at the family level in neonates with jaundice group, which may be jaundice-preventive because they inhibit β-glucuronidase, thereby accelerating the deconjugation of conjugated bilirubin in the gut. Neonates with jaundice develop a gut imbalance characterized by decreased abundance of *Bifidobacteriales* [35]. Gut microbiota was in a state of dysbiosis and significantly lower levels of *Bifidobacteriales* were observed at the discovery stage in children with autism spectrum disorders (ASD). An increase in *Bifidobacteriales* was related with significant reduction in the severity of ASD and gastrointestinal symptoms [36].			*	*
*Actinobacteria*	*Actinobacteria* are Gram-positive bacteria containing a GC-rich linear genome with the robust biosynthetic potential to produce secondary metabolites of broad structural diversity [37]. *Actinobacteria* are the source of all naturally derived antibiotics and a range of anticancer and immunosuppressive drugs.More new antibiotics may be found from actinomycetes. These compounds may become powerful treatment and stimulate the chemical synthesis of new compounds [38].				*
*Holdermania*	*Holdemania* were enriched in the feces of (Parkinson’s disease, PD) patients after adjusting for age, gender, body mass index (BMI), and constipation [39]. *Holdemania* was decreased in the general adult population who took the multi-strain probiotics. The decrease in *Holdemania* following supplement administration suggest that assessing the potential positive impacts on obesity or metabolic disease is warranted [40].		*	*	
*Proteus*	*Proteus* spp. are Gram-negative bacteria belonging the Enterobacteriaceae family. Proteus is an independent risk factor for diabetes [41]. The identification of *Proteus* spp. as potential pathogens in Crohn’s disease recurrence after intestinal resection serves as a stimulus to examine their potential role as gut pathogens. *Proteus* species are low-abundance commensals of the human gut that harbor significant pathogenic potential [42]. *Proteus* species have been associated with infectious gastroenteritis [43]. Patients with cirrhosis had an elevated proportion of *Proteus* species compared with people with non-cirrhosis. In the hepatobiliary tract, *Proteus* spp. are an uncommon cause of infection and are usually related to surgical interventions, such as endoscopic retrograde cholangiopancreatography (ERCP) or abdominal surgery [44].		*	*	
Aeromonadales	*Aeromonadales* is classified as aerobic and facultative anaerobes, which can cause many diseases such as enteritis and sepsis and is also related to kidney and cardiovascular problems. A study showed that Shenqi Yanshen Formula (SQYSF) significantly reduced the degree of renal fibrosis in chronic kidney disease mice and greatly increased the abundance of *Aeromonas* in the intestinal tract of mice [45].	*			
*Desulfovibrio*	Nitrogen fixation can occur in the human gut. *Desulfovibrio* diazotrophicus is a sulfate-reducing bacterium from the human gut that can fix nitrogen [46]. High abundance of *Desulfovibriob* as a gramnegative bacteria in people with depression may explain the contribution of microbiota in development of depression [47].	*			
*Leuconostocaceae*	Human studies demonstrated the lower abundance of *Leuconostocaceae* in persons with depression [48].	*			
*Weissella*	*Weissella* strains can control foodborne pathogens because they can produce bacteriocins, hydrogen peroxide and organic acids; *Weissella* has also shown potential to treat atopic dermatitis and certain cancers. Animal studies have shown that *Weissella* strains contribute to the recovery of lymphocyte, hemoglobin and platelet levels, *Weissella* strains have also been shown to be effective in the treatment of atopic dermatitis. Further exploration is needed to determine the effects of *Weissella* strains on human health [49].	*			
*Prevotella*	In the human microbiome, *Prevotella* spp. are highly abundant in various body sites, where they are key players in the balance between health and disease. *Prevotella* is associated with inflammatory autoimmune diseases, bacterial vaginosis and other diseases. At present, the direct cause of the disease is uncertain. The effect of *Prevotella* on health is unclear, and its relationship with glucose homeostasis is also inconsistent [50].	*			
*Gemella*	*Gemella* is gastrointestinal microbiota, gram-positive cocci that behave like viridans group streptococci. Despite the low incidence of bacteremia from these organisms, they can lead to infective endocarditis (IE) and other clinical syndromes. The level of *Gemella* should be comprehensively checked when infective endocarditis occurs [51].	*			
*Carnobacteriaceae*	People who consumed more ultra-processed foods (UPFs) presented an increase of *Carnobacteriaceae*, which has been also related to obesity [52].		*		
*Granulicatella*	*Granulicatella* is a type of nutritionally variant Streptococcus (NVS) that requires special medium for growth [53]. *Granulicatella* was more abundant in subjects with a high inflammatory index [54].		*		

“*” means higher level of abundance.

## Discussion

The changes in the gut microbiome in pregnant women with GDM before and after MNT have not been studied. The features of the gut microbiome in pregnant women with GDM whose glycemic control was effective and ineffective were investigated and compared in this study to discover target gut microbiome that may influence the efficiency of MNT glycemic control. The findings revealed that lower *Oscillatoria* abundances and higher Bifidobacterium abundances were advantageous to the effect of glycemic management in pregnant women with GDM.

The crucial role of the gut microbiome in modulating insulin resistance and the inflammatory response in pregnant women with GDM has been reported by a few studies [55,56]. Experiments have shown that compared to normal pregnant women, pregnant women with GDM have an imbalanced gut microbiome that leads to increased absorption of glucose and fatty acids, and further exacerbates the inflammatory state of pregnant women through the immune system, thereby increasing the level of insulin resistance. On one hand, the metabolic consumption of the body as well as the demand for nutrients increases with gestational age, and pregnant women are autonomous to consume more food to supplement, including high calorie foods rich in carbohydrates and fats during pregnancy, which will make it difficult for pregnant women with GDM to control their blood glucose levels. On the other hand, previous studies have found that metformin treatment of GDM can reduce newborn birth weight in a short period of time, but the long-term weight gain of children is faster than that of the insulin treatment group, and women with GDM who take metformin were more likely to gain less than the recommended amount of weight during pregnancy [57,58]. Due to the imbalance of the gut microbiome and the risk of oral medications, it is still difficult for pregnant women with GDM to achieve the glycemic control standard, and it is important to explore safer and more effective way to help control blood glucose levels based on MNT.

Up till the present moment, the efficacy of MNT on glycemic control in pregnant women with GDM is affected by multiple factors, and it is difficult to take into account the assessment errors present in the individual life environment of pregnant women. To improve the effectiveness of MNT, investigators have performed laboratory and population-based studies from the gut microbiome module associated with GDM to further improve glycemic control in pregnant women with GDM [59]. In recent years, numerous scholars have assisted the MNT of pregnant women with GDM from the perspective of probiotic therapy. A meta-analysis evaluating the safety of probiotic intake during pregnancy found that taking probiotics or prebiotics during pregnancy did not affect the risk of preterm birth or cause adverse effects on the mother or baby. A randomized double-blind controlled experiment found that the FBG of pregnant women taking probiotics was significantly higher than that of the placebo group [60]. The results of another randomized double-blind controlled trial found that supplementing with probiotics could not improve blood glucose, insulin and other related indicators [61]. Nevertheless, other findings suggest that probiotics can improve the glucose metabolism of pregnant women at the same time [62]. Therefore, it is controversial whether the administration of probiotics or prebiotics during pregnancy can improve the blood glucose level of pregnant women with GDM. The reasons for these inconsistent results may be the inconsistent use of probiotics, the presence of a single probiotic or a combination of multiple probiotics, and the different strains of interventions in the current studies [63].

Our study found that pregnant women with GDM in the effective group had higher changes in alpha and beta diversity indicators of gut microbiome compared to the ineffective group before and after the MNT, a trend that was not statistically significant. The OGTT results in this study showed that the one-hour postprandial blood glucose of the ineffective group was significantly higher than in the effective group, and there was also a trend towards higher FBG and 2-hour postprandial blood glucose, which to some extent suggested that the blood glucose level was affected by the diversity of gut microbiome. However, this may be due to the small sample size of the final test and the fact that the subjects in this study were all pregnant women with GDM by matching the age and BMI, which did not discriminate to a significant degree in comparison to healthy pregnant women. The difference in results was not statistically significant.

We found characteristic microbiome with obvious differences in two groups before MNT. The gut microbiome of pregnant women in the effective group was richer in *Rosella*, *Bifidobacterium*, *Clostridium*, *Holdemania* and *Proteus* before the MNT. It has been proved that *Holdemania* and *Proteus* are significantly related to the increase of blood glucose caused by impaired glucose metabolism in the body [64–66]. Meanwhile, *Rosella*, *Bifidobacterium* and *Clostridium* have been found to be beneficial, improving blood glucose levels and reducing inflammation response and insulin resistance [13,67–70]. In the ineffective group, there were also enriched bacteria in the intestine such as *Leuconostocaceae*, *Weissella*, *Prevotella*, and *Bacillus cereus* [71,72] that help the body improve blood glucose level. Harmful bacteria such as *Desulfovibrio*, *Aeromonas* and *Gemella* that result in body weight gain, impaired glucose tolerance and insulin resistance [73–76] were enriched in the ineffective group. It is reasonable for pregnant women with GDM to accumulate the gut microbiome positively or negatively related to blood glucose level before MNT, which may result from the pregnant women themselves suffering from GDM. The accumulation of microbiome in the gut that caused inflammation response and impaired glucose tolerance as well as insulin resistance was within acceptable limits compared to healthy pregnant women.

Before and after MNT alone, the two groups were observed for changes in their own gut microbiome. There were no differences in gut microbiome of the ineffective group, however, the proportion of *Oscillatoria* in the intestinal tract decreased significantly in the effective group after MNT. Studies have found that *Oscillatoria* is involved in the decomposition and fermentation of proteins in the intestine, and this process will produce toxic metabolites which also lead to the proliferation of conditional pathogens and pro-inflammatory bacteria, thus affecting the body’s blood glucose level [77]. Therefore, it is possible that the effective group of pregnant women also have a reduction in pro-inflammatory bacteria in their gut due to a significant reduction of *Oscillatoria*, which can reduce the body’s inflammation level and improve insulin resistance, thereby improving blood glucose levels.

The analysis and comparison of species differences in the gut microbiome of the two groups after MNT showed that the effective group was enriched in *Actinobacteria* and *Bifidobacteria*. *Actinomycetes* were positively correlated with insulin resistance [67], and the enrichment of *Bifidobacterium* can improve the blood glucose level of pregnant women. However, the ineffective group was enriched in *Holdemania* and *Proteus* in the gut, which were obviously associated with impaired glucose metabolism and elevated blood glucose levels in vivo. *Carnobacteriaceae* and *Granulicatella* were also enriched in the ineffective group. Studies have found that *Carnobacteriaceae* can release endotoxins to cause the body’s inflammatory state, and *Granulicatella* has been shown to be clearly related to the occurrence of intestinal tract inflammation [78]. This difference may explain the failure to control blood glucose in pregnant women with GDM and suggest that pregnant women with GDM can be given *Bifidobacteria*-based probiotics or prebiotics to help them control their own blood glucose levels during the one-week MNT phase. Real-world evidence are considered in future studies to include *bifidobacteria*-based probiotic or prebiotic interventions to further clarify whether improving the gut microbiome or oral probiotics is effective in improving blood glucose levels in humans. The results of this study found that the blood glucose level was negatively correlated with gut microbiome such as *Leuconostocaceae*, *Weissella*, *Pseudomonas*, *Bacillales_Incertae Sedis XI*, *Gemella* and *Bacillales*, and the potential mechanism need to be investigated. In later studies, we can further dig into the role of the above gut microbiome in the regulation of blood glucose levels in humans.

This is a first study that focused on the changes in gut microbiome characteristics of pregnant women with GDM before and after MNT, and the relationship between the gut microbiome characteristics and the effect of MNT on glycemic control. There are still some limitations in our study. First, gestational age was judged according to the time of the last menstruation period and ultrasound (Nuchal Translucency time, NT), mainly according to the time displayed by ultrasound. If the results obtained by the two methods differ by more than one-week, gestational age calculation will favor the time displayed by the NT. However, these two methods are not perfect, and we will further optimize the way of evaluating gestational age in the future. Second, the participants in this study were recruited from a single obstetrics clinic, the results may not be generalizable to a broader population of women with GDM. In future studies, we will conduct multicenter studies with expanded sample size to further elucidate the role of the gut microbiota and its relationship with GDM. Third, compared with the current clinical MNT, the present study MNT has higher requirements for the study subjects. MNT ended up in the form of recipes and was difficult to implement, which led to low adherence and easy loss to follow-up, thus reducing the sample size of this study. In addition, it is imperative to develop a more adaptive MNT regimen with the addition of *Bifidobacterium* dominated probiotic or prebiotic intervention. In conclusion, this study explored the relationship between gut microbiome characteristics and glycemic control effect before and after MNT in pregnant women with GDM, in order to provide a new reference basis and method for effectively improving glycemic control effect of MNT in clinical pregnant women with GDM from the perspective of gut microbiome.
